# The dilemma of frontline servant leadership: The conflict between top management directives and grassroots employee wellbeing and retention

**DOI:** 10.1371/journal.pone.0323811

**Published:** 2025-05-14

**Authors:** Zhiwen Song, Ziyu Xu, Binglin Martin Tang, Wai Nga Leong

**Affiliations:** 1 Faculty of International Tourism and Management, City University of Macau, Taipa, Macau, China; 2 Faculty of Business, City University of Macau, Taipa, Macau, China; 3 Faculty of International Tourism and Management, City University of Macau, Taipa, Macau, China; 4 Faculty of Business, City University of Macau, Taipa, Macau, China; Federal University of Santa Maria: Universidade Federal de Santa Maria, BRAZIL

## Abstract

Servant leadership in the workplace involves providing services and cultivating trust amongst subordinates. However, leadership dynamics can be distorted when hospitality frontline managers assume leadership roles by strictly adhering to top management strategic directives. This study aims to examine how constraints imposed by top management influence the management styles of frontline managers within the hospitality industry. The current study delved into the interplay amongst servant leadership, employee wellbeing and retention, highlighting the pivotal role of service climate, procedural justice and customer satisfaction as moderating variables. In this research, partial least squares structural equation modelling was used to test the research model, using data collected from 485 respondents. Findings underscore the critical role of hospitality frontline managers in aligning with organisational strategic directives, a requirement that may compromise their servant leadership style. Poor service climate and procedural justice may result from this alignment. The current study emphasises that strictly adhering to predetermined top management decisions can influence the outcomes of servant leadership—an often-overlooked factor in existing research. Lastly, this research enriches the literature on the effect of servant leadership on employee retention.

## 1. Introduction

Hospitality is a labour-intensive industry [1], and the wellbeing and retention of employees is critical to business operations and performance. Generally, the voluntary turnover rate in the hotel industry is always higher than that in other sectors, typically ranging from 30% to 300%. In China, the average turnover rate across all industries is about 20%, whilst the voluntary turnover rate in the hotel industry reaches as high as 40%, which is the highest in the country [2]. The hospitality industry faces high employee turnover and poor employee wellbeing [3], thereby posing a challenge to the sustainable development of businesses [4,5].

Empirical studies within the hospitality industry focus on the correlation between frontline managers and their leadership styles. However, a significant gap exists on the influence of top management on the service climate and procedural justice, thereby impacting the leadership attitudes and behaviour of hospitality frontline managers [6–8].

Given that the focus shifts towards compliance with top management directives, a growing body of research has explored how servant leadership influence employees’ job satisfaction, health wellbeing and loyalty to the organisation [9,10]. However, the impact of top management’s constraints on servant leadership and its subsequent effect on employee retention remains underexplored. When frontline managers are unable to practice servant leadership owing to top management’s directives, it can lead to decreased employee wellbeing and increased turnover intentions [9,11]. Studies have found that the suppression of servant leadership can lead to a decline in managerial performance and disrupt daily organisational operations [9]. This decline in performance can further impact employee wellbeing and retention because employees may feel unsupported and undervalued.

Notably, employee satisfaction and retention are also influenced by the organisational climate [12]. Hospitality industry problems often exist and caused by top management failing to utilise organisational resources to meet frontline managers and employees’ needs [13]. Although frontline hotel managers have certain freedom to implement a servant leadership style in practice, they still need to follow the organisational norms and standards of practice set by top management. Many research empirical findings exhibit hospitality support, possibly affecting leadership style in achieving negative or positive results [14]. However, such findings disregard the idea that service climate and procedural justice can influence managers’ leadership approach and their ability to cultivate trust amongst their employees [15].

Previous studies [16–18] have discussed servant leadership within the hospitality sector and the hindrances posed by the service environment under top management [19–21]. However, some studies have focused on leadership styles’ positive aspects, overlooking such antecedents as top management norms and organisational decisions, which may cause frontline managers to misinterpret management styles [22]. Furthermore, previous studies [23–25] have also confirmed that procedural fairness affects employees’ job satisfaction and their decisions to stay or leave a job, which is related to leader behavior [26,27]. Therefore, this study explores how top managers’ influence on frontline managers alters their management approach and perceptions of service climate and procedural justice, and how these changes affect their servant leadership style [28–30].

The guidance provided by top management influences service climate and procedural justice within organisations, thereby shaping leadership styles [7,31,32]. Moreover, organisational cues emphasising additional job demands and expectations from top management could diminish frontline managers’ leadership efficacy and conduct [9]. According to early social learning theory [33], employees tend to emulate the work behaviour of role models in the work environment. Therefore, frontline managers likely emulate the attitudes and behaviours of top management, integrating them into the organisation’s acceptable work attitudes.

This study uses top management decision-making as a context and examines how frontline servant leadership, moderated by service climate and procedural justice, affects the wellbeing and retention of junior employees. The results show that frontline managers’ servant leadership style may be compromised by top management’s restrictions. This compromise could eventually affect employees’ wellbeing and retention. This study fills in the aforementioned research gap.

This study provides the following objectives to guide this research:

(1) reveal the relationship between servant leadership and employee wellbeing and retention as measured in the service industry;
(2) investigate whether or not service climate and procedural justice serve as a moderator in the relationship between servant leadership and employees’ wellbeing; and
(3) explore whether or not customer satisfaction serves as a moderator in the relationship between employee wellbeing and retention.

The current study delves into three messages, namely, contributing knowledge of management, psychological studies and human resources management, thereby enhancing the overall value of this research. Theoretical insights are refined by exploring the impact of servant leadership dimensions on employee wellbeing and retention based on leader–member-exchange (LMX) theory and role theory. By integrating and extending these theories, this study advances the dissemination of leadership knowledge and strengthens the foundation of servant leadership research, thereby filling in existing gaps in the field.

## 2. Literature review and hypothesis

### 2.1. LMX and role theories

LMX theory and role theory guided this article [34,35]. LMX theory lies in the concept that leaders form trusting, solid, emotional and respect-based relationships with specific team members, potentially increasing interpersonal relationships [36]. Note that LMX theory, which emphasises the quality of relationship between leaders and members and its impact on employee behavior and attitudes, is highly relevant to this study’s focus on the impact of servant leadership on employee wellbeing and retention. Many studies have underscored the need to enhance employee satisfaction through leadership practices. This enhancement can ultimately contribute to employee turnover rate reduction [37–39]. Some researchers have also indicated that servant leadership serves as a mechanism to cultivate organisational citizenship, strengthen commitment and reduce high turnover from the LMX theory perspective [40].

Biddle [41] firstly introduced role theory from cognitive social psychology. The ‘roles’ represent the share of normative expectations and define particular social positions and corresponding behaviour [41]. As the concept of servant leadership evolves, frontline managers assume leadership roles and also adhere to the strategic guidance provided by top management, thereby integrating their management style into the organisational framework. Frontline managers adopt their social norms and special roles based on cues from top management’s expectations [9]. Frontline managers differ from top management and come in various types. The latter may sacrifice employees’ wellbeing and decision-making for their own benefits [42]. Role theory can explain how leadership behaviours can influence employees’ perceptions and behaviours, especially under top management constraints, in which servant leaders may be unable to maximise their positive roles. Therefore, we anticipate that role theory will be the most suitable framework for incorporating our proposed model. This theory can effectively justify how service climate and procedural justice are associated with servant leadership.

### 2.2. Top management and servant leadership

Top management comprises senior executives and managing directors managing organisations [22,43]. They manipulate the service climate and procedural justice and shape frontline managers’ leadership style. They create visibility and power for the followers. Therefore, we propose top management norms and code of ethics and mould the service climate and justice in organisations that help interpret and develop the leadership style of frontline managers [44]. However, top management can also pressure frontline leaders [45].

Servant leadership is one type of frontline leadership. Canavesi and Minelli [46] defined servant leadership as a leadership philosophy and practice centred on prioritising the interests of those led over the leaders’ own. This leadership underscores the importance of leaders’ actions being geared more towards fostering the gains of subordinates than leaders’ concerns. Servant leadership entails assisting subordinates, establishing trust and inspiring them to follow leaders [8]. Corresponding with LMX theory [36], offering motivational and supportive resources to employees is an important component of increasing employee engagement. This importance coincides with the management characteristics of servant leadership. Once employees feel invested in their leaders at work, they become more loyal to organisations and less inclined to leave.

Leader personality is positively correlated with employees’ perceptions of an atmosphere of procedural justice [47], and employees use procedural justice as a signal to determine whether or not their leaders value them [48]. However, low servant leadership may change their perception of the followers rather than serve to achieve the organisations’ mission [49]. Therefore, this study chose servant leadership as the research object.

### 2.3. Overview of hypothesis development

Relevant leadership can provide meaningful support for employees’ basic psychological needs for autonomy, competence and relatedness to promote employees’ self-regulation and satisfy followers’ autonomy needs through empowerment [31,50].

Empirical research has highlighted that wellbeing psychology links to employees’ satisfaction and organisations’ management of emotionally healthy workplaces [51]. Servant leaders’ emotionally healing behaviours can also satisfy followers’ need for relatedness, thereby enabling them to feel understood and appreciated by recognising and connecting with their situations and emotional distress [52]. Conversely, low servant leadership may affect employees’ wellbeing.

Therefore, this study proposes the following hypothesis

*Hypothesis 1 (H1): Low servant leadership has a negative effect on employees’ wellbeing.*

Extensive evidence has shown that employee wellbeing affects a variety of performance indicators, including productivity, employee turnover, job satisfaction, job security, rewards and recognition, stress and work–life balance [53,54]. These indicators are related to the antecedents of employee retention [53]. DiPietro et al. [55]showed that workplace wellbeing significantly affects turnover intentions when moderated by positive affective commitment, negative affective commitment; and job satisfaction when moderated by positive affective commitment. Moreover, workplace wellbeing significantly affects turnover intentions. This finding relatively confirms the model of employee wellbeing factors and employee retention rate proposed by Gelencsér et al. [56]. Therefore, employee wellbeing can be a corporate contributor to employee retention. This study proposes the following hypothesis based on the findings on employee wellbeing and employee retention:

*Hypothesis 2 (H2): Low employee wellbeing has a negative effect on employee retention.*

Service climate refers to the service-focused policies, practices and procedures people experience in their work units and the behaviours they observe as being rewarded, supported and expected [57]. This climate comprises interactions amongst employees, and the format and quality of these interactions are the basic manifestations of forming a service atmosphere. A discussion exhibits the consequences of attitudes, organisational citizenship behaviour and service performance influenced by service climates [58,59]. If the organisation performance and climate are weak, then leaders perceive negative supportive service organisations, which will affect their performance and employee satisfaction. Conversely, strong and positive service climates may share the perception of reward and support and can predict positive outcomes [60]. Veld and Alfes [61] confirmed that employee wellbeing can be improved by creating a happy atmosphere. Conversely, poor service climate affect servant leadership and employee wellbeing. Therefore, this study proposes the following hypothesis:

*Hypothesis 3 (H3): Service climate serves as a moderator in the relationship between servant leadership and employee wellbeing.*

Procedural justice refers to the perception that the processes and procedures used by organisations to achieve significant results are fair and justice [62,63]. This type of justice is part of the multidimensional justice structure, including distributive, procedural, informational and interpersonal justice [64]. Leader personality is positively related to employees’ perceptions of procedural justice climate [65], and employees will use procedural justice as a signal to judge whether or not leaders value them [66]. Frontline managers adopt the request from top management; complying with organisations’ expectations may harm employees’ well-being. Therefore, procedural injustice may be related to low servant leadership and employees’ psychological wellbeing.

The relationship between procedural justice and employee wellbeing is considered across various disciplines [67]. A link has been found between employees’ perceptions of procedural injustice and different health problems after experiencing chronic stress processes. The lack of control over procedures, rules, work and decision-making processes will increase employee anxiety, thereby negatively impacting employee wellbeing [68,69]. Therefore, this study proposes the following hypothesis:

*Hypothesis 4 (H4): Procedural justice is a moderator in the relationship between servant leadership and employee wellbeing.*

Customer satisfaction refers to the degree of satisfaction customers express after the service delivery process [70]. When service providers are highly committed to their role in the service process, they are likely to be motivated to continue to provide satisfactory service to customers. Frey et al. [71] showed that customer satisfaction significantly impacts the satisfaction and subjective wellbeing of corporate employees and is an important factor in improving employee retention rates. Therefore, this study proposes the following hypothesis:

*Hypothesis 5 (H5): Customer satisfaction is a moderator in the relationship between employee wellbeing and employee retention.*

**Fig 1:**
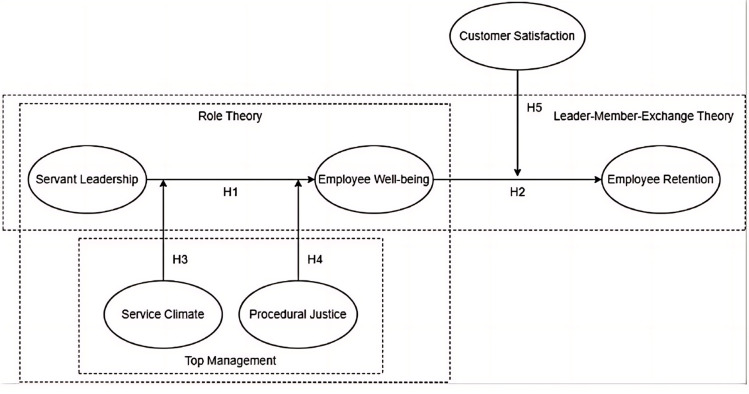
Research model.

The proposed hypotheses form the research model, as shown in Fig 1.

## 3. Methods

### 3.1. Participants and design

This study was designed using a quantitative research method. Quantitative methods are most conducive to testing existing theories and hypotheses, which can quantify the research objects and correlations within them [72]. Accordingly, this study used the Servant Leadership Measurement Scale and the Employee Well-being Measurement Scale that other researchers constructed and validated [73,74]. This research adopted judgemental sampling and collected data from the hospitality industry’s frontline employees in China [75]. The respondents primarily represent hotel frontline departments. The questionnaire was distributed from 1 June to 31 August 2024. The questionnaire was distributed to the human resources departments through personal connections and delivered to the respondents. All respondents signed a written form of informed consent and participated in this survey after understanding the purpose of the study and their rights. This questionnaire is anonymous because it focuses on employees’ views on companies’ leadership and career plans. This study has been approved by the Ethics Committee of the City University of Macao. The research procedure follows the purpose of the Declaration of Helsinki.

The questionnaire contents adopted back translation to provide credibility [76]. A total of 550 questionnaires were sent, 509 of which were collected, for a response rate of 92.5% [77]. The total of valid questionnaires was 485, and the effective questionnaire response rate was 88%. We adopted Smart-PLS4 software to explore the relationship between constructs.

### 3.2. Measures

This study adopted well-developed scales to measure all the aforementioned constructs. A five-point Likert scale was used to evaluate each construct.

#### Servant leadership.

Van Dierendonck [78] developed the servant leadership measurement scale, which comprises eight dimensions: empowerment, support, responsibility, understanding, courage, authenticity, humility and stewardship. The current study focused solely on the leadership perceptions of frontline managers, rather than those of other individual employees. This focus is justified by the findings of Ling [79], demonstrating that the leadership style of frontline managers has the most significant impact on frontline employees compared with the influence of leaders at other organisational levels, such as top-level leaders.

#### Employee wellbeing.

Zheng et al. [74] and Becker et al. [73] referred to a previous scale and divided employee wellbeing into three dimensions.

#### Employee retention.

This study mainly drew on the employee turnover intention and employee retention scales developed by DiPietro et al. [80] and Arasanmi and Krishna [81], comprising five items.

#### Service climate.

This study used the service climate measurement scale developed by Ashkanasy et al. [82] and Kralj and Solnet [83], comprising six items.

#### Procedural justice.

The scale measuring procedural justice was modified based on Erdogan et al. [84] and Ehrhart [47]. A total of four item scales were adopted in this research.

#### Customer satisfaction.

This study adopted the customer satisfaction measurement scale developed by Chan et al. [85] and Huang et al. [86], comprising four items.

## 4. Data analysis

In this study, common method bias (CMB) was detected using Harman’s single-factor test. The result of the variance explained by the first principal component extracted without rotation was 35.57%, which was 50% less than the threshold, indicating no serious CMB problem in this study [87].

Table 1 shows that demographic details from 485 valid samples are included in the data analysis. A total of 303 women (62.5%) and 182 men (37.5%) participated in the survey. The respondents are mainly 26–35 years old (75.1%). The education levels are mainly undergraduate and master’s degrees (78.6%). Working experience is usually one to three years (66.4%). Descriptive data analysis is presented in Table 2.

**Table 1:** Demographic Information.

	Categories	Frequencies	Percentages
Gender	Male	182	37.5%
	Female	303	62.5%
Age	18–25 years old	364	75.1%
	26–35 years old	26	5.4%
	36–45 years old	34	7.0%
Education level	46–55 years old	52	10.7%
	Over 55 years old	9	1.9%
	High school and below	24	4.9%
	Junior college	43	8.9%
	Undergraduate	194	40.0%
	Master’s	187	38.6%
	Doctoral	37	7.6%
Years of work	1–3 years	322	66.4%
	4–6 years	40	8.2%
	7–10 years	26	5.4%
	Over 10 years	97	20.0%
Job ranking	Operation level	219	45.2%
	Supervisory level	35	7.2%
	Managerial level	106	21.9%
	Others	125	25.8%
Total		485	100%

^2^N = 485.

**Table 2:** Descriptive data analysis.

Names	Mean	Median	SD	EK	Skewness
EP1	1.895	2.000	0.807	0.960	0.832
EP2	1.973	2.000	0.843	1.085	0.879
EP3	1.990	2.000	0.834	−0.035	0.555
EP4	2.031	2.000	0.867	0.141	0.569
SB1	2.781	3.000	1.073	−0.742	0.043
SB2	2.827	3.000	1.041	−0.712	−0.056
SB3	2.301	2.000	0.854	−0.473	0.216
SB4	2.016	2.000	0.812	−0.263	0.479
AC1	2.586	2.000	1.006	−0.665	0.279
AC2	2.428	2.000	0.924	−0.411	0.338
AC3	2.457	2.000	0.932	−0.001	0.424
FG1	2.363	2.000	0.968	−0.250	0.480
FG2	2.447	2.000	0.955	−0.396	0.286
FG3	2.538	2.000	0.895	0.184	0.422
CO1	2.913	3.000	1.028	−0.745	0.094
CO2	2.464	2.000	0.927	−0.691	0.168
CO3	2.895	3.000	1.088	−0.533	0.046
AU1	2.487	2.000	0.868	−0.117	0.326
AU2	2.365	2.000	0.850	−0.462	0.336
AU3	2.355	2.000	0.971	−0.221	0.448
HU1	2.291	2.000	0.842	−0.109	0.239
HU2	2.122	2.000	0.804	0.754	0.610
HU3	2.136	2.000	0.767	−0.267	0.287
HU4	2.247	2.000	0.828	0.584	0.583
ST1	1.946	2.000	0.766	−0.328	0.423
ST2	2.074	2.000	0.868	−0.323	0.444
ST3	2.146	2.000	0.904	0.465	0.714
SC1	2.920	4.000	0.804	0.506	0.497
SC2	2.887	4.000	0.813	0.049	0.458
SC3	2.889	4.000	0.849	0.462	0.636
SC4	2.967	4.000	0.880	0.187	0.628
SC5	2.955	4.000	0.841	0.542	0.707
SC6	1.019	2.000	0.867	0.600	−0.744
LWB1	3.235	3.000	0.994	−0.456	−0.169
LWB2	3.515	3.000	0.872	0.021	−0.178
LWB3	3.278	3.000	0.950	−0.546	−0.002
WWB1	3.825	4.000	0.840	−0.457	−0.267
WWB2	3.410	3.000	0.894	−0.198	−0.120
WWB3	3.736	4.000	0.830	−0.173	−0.320
PWB1	3.938	4.000	0.689	−0.016	−0.261
PWB2	3.794	4.000	0.794	−0.100	−0.260
PWB3	3.738	4.000	0.768	−0.154	−0.200
PJ1	3.678	4.000	0.812	−0.267	−0.252
PJ2	3.511	4.000	0.819	0.050	−0.172
PJ3	3.625	4.000	0.829	0.657	−0.684
PJ4	3.720	4.000	0.797	0.187	−0.386
CS1	3.767	4.000	0.803	−0.255	−0.345
CS2	3.810	4.000	0.769	1.031	−0.618
CS3	3.924	4.000	0.741	−0.046	−0.366
CS4	3.825	4.000	0.698	0.018	−0.257
ER1	3.569	4.000	0.949	0.229	−0.598
ER2	3.699	4.000	0.805	0.124	−0.497
ER3	3.546	4.000	0.963	0.077	−0.583
ER4	3.753	4.000	0.860	0.861	−0.771
ER5	3.567	4.000	0.941	0.333	−0.754

^3^Note: SD = Standard deviation; EK = Excess kurtosis.

### 4.1. Reliability, validity and correlation

Table 3 presents the reliabilities, factor loadings (FL), average variance extracted (AVE) and composite reliabilities (CR) for the constructs in this study. All Cronbach’s alpha values, which are for the internal consistency examinations, are above 0.7.

**Table 3:** Reliability and validity of the constructs.

Scale items	Abbreviations	Factor loadings	Cronbach’s α	CR	AVE
Servant leadership (including eight dimensions)					
Empowerment			0.830	0.836	0.662
My manager does not encourage me to use my talents.	EP1	0.989			
My manager does not encourage his/her staff to come up with new ideas.	EP2	0.958			
My manager does not just tell me what to do but enables me to solve problems myself.	EP3	0.984			
My manager does not offer me abundant opportunities to learn new skills.	EP4	0.866			
Standing back			0.783	0.797	0.821
My manager does not keep himself/herself in the background and gives credits to others.	SB2	0.880			
My manager appears to enjoy his/her own success more than his/her colleagues’ success.	SB3	0.865			
My manager does not encourage me to handle important work decisions on my own.	SB4	0.840			
Accountability			0.717	0.718	0.779
My manager does not hold me responsible for the work I carry out.	AC1	0.848			
I am not held accountable for my performance by my manager.	AC2	0.892			
My manager does not hold me and my colleagues responsible for the way we handle a job.	AC3	0.858			
Forgiveness			0.762	0.772	0.676
My manager does not keep criticising people for the mistakes they have made in their work (r)	FG1	0.903			
My manager does not maintain a hard attitude towards people who have offended him/her at work (r).	FG2	0.846			
My manager finds it is not difficult to forget things that went wrong in the past (r).	FG3	0.840			
Courage			0.749	0.763	0.662
My manager does not take risks when he/she is not certain of the support from his/her own manager.	CO1	0.826			
My manager does not take risks and does what needs to be done in his/her view.	CO2	0.872			
My manager is unwilling to risk rejection by someone important to get the opportunity to fulfill my life goals tome.	CO3	0.843			
Authenticity			0.773	0.783	0.687
My manager is not open about his/her limitations and weaknesses.	AU1	0.862			
My manager is not prepared to express his/her feelings even if this might have undesirable consequences.	AU2	0.807			
My manager does not show his/her true feelings to his/her staff.	AU3	0.803			
Humility			0.799	0.820	0.623
My manager does not learn from criticism.	HU1	0.797			
My manager does not try to learn from the criticism he/she gets from his/her superior.	HU2	0.804			
My manager does not admit his/her mistakes to his/her superior.	HU3	0.880			
My manager does not learn from the different views and opinions of others.	HU4	0.825			
Stewardship			0.746	0.746	0.663
My manager does not emphasise the importance of focusing on the good of the whole.	ST1	0.852			
My manager does not have a long-term vision.	ST2	0.826			
My manager does not emphasise the societal responsibility of our work.	ST3	0.847			
Service climate			0.895	0.895	0.704
Company management supports employees when they come up with new ideas on improved customer service.	SC1	0.814			
Company management sets definite quality standards of good customer service.	SC2	0.859			
Company management meets regularly with employees to discuss performance goals.	SC3	0.857			
New employees are trained by company management on how to best serve customers.	SC4	0.913			
Company management continually communicates the importance of service.	SC5	0.881			
There is a true commitment to service, not just ‘lip service’.	SC6	0.876			
Subjective wellbeing (including three dimensions)					
Life wellbeing			0.842	0.844	0.759
I am close to my dreaming most aspects of my life.	LWB1	0.841			
Most of the time, I do feel real happiness.	LWB2	0.840			
So far, I have gotten the important things I want in life.	LWB3	0.848			
Work wellbeing			0.724	0.725	0.644
I am satisfied with my work responsibilities.	WWB1	0.871			
I find real enjoyment in my work.	WWB2	0.863			
I can always find ways to enrich my work.	WWB3	0.834			
Psychological wellbeing			0.718	0.724	0.640
I handle daily affairs well.	PWB1	0.845			
I generally feel good about myself, and I am confident.	PWB2	0.778			
People think I am willing to give and to share my time with others.	PWB3	0.895			
Procedure justice			0.830	0.838	0.661
I believe my manager really tries to conduct a fair and objective appraisal.	PJ1	0.852			
Is procedural justice free of bias in your department?	PJ2	0.864			
Are your department’s procedures consistent with ethical standards?	PJ3	0.883			
Are your department’s procedures consistent with ethical standards?	PJ4	0.869			
Customer satisfaction			0.877	0.879	0.731
I am satisfied with the service we provide.	CS1	0.894			
Our internal operations are efficient and keep our customers satisfied.	CS2	0.849			
The attitude of our staff makes customers satisfied.	CS3	0.801			
Did your customer service experience meet your expectations?	CS4	0.834			
Employee retention			0.871	0.881	0.658
I feel satisfied with my work in this organisation.	ER1	0.895			
Level of satisfaction with job	ER2	0.867			
Experience: Advancement Opportunities	ER3	0.837			
If I wanted to move into another job or function, then I would firstly look within the organisation for possibilities.	ER4	0.871			
If it were up to me, then I would definitely be working for this agency for the next five years	ER5	0.855			

To assess construct validity, all items’ factor loadings should be above 0.7. Otherwise, unqualified items should be removed [88]. Thus, SB1, SB2, AC1 and SC6 4 were removed for further analysis. The AVE values should be above 0.5, and CR should be over 0.7. Therefore, the results align with the guidelines set by Hair et al. [88], and the current study’s internal consistency reliability and validity are satisfactory.

As shown in Table 4, the square root of each construct’s AVE was greater than its correlation with the other constructs. Moreover, the HTMT value of all constructs was below 0.85. These results confirm the discriminant validity between all constructs [89]. Therefore, the results align with the reliability and validity test guidelines set by Hair et al. [88].

**Table 4:** Discriminant test.

	AC	AU	CO	CS	EP	ER	FG	HU	LWB	PJ	PWB	SB	SC	ST	WWB
AC	**0.977**														
AU	0.614 (0.514)	**0.870**													
CO	0.679 (0.435)	0.694 (0.769)	**0.860**												
CS	0.544 (0.418)	0.620 (0.548)	0.557 (0.533)	**0.862**											
EP	0.607 (0.503)	0.565 (0.451)	0.512 (0.528)	0.578 (0.478)	**0.851**										
ER	0.539 (0.439)	0.577 (0.577)	0.601 (0.620)	0.621 (0.680)	0.528 (0.541)	**0.819**									
FG	0.873 (0.726)	0.677 (0.635)	0.672 (0.787)	0.565 (0.518)	0.629 (0.689)	0.544 (0.550)	**0.834**								
HU	0.560 (0.490)	0.721 (0.725)	0.571 (0.604)	0.652 (0.662)	0.575 (0.623)	0.544 (0.547)	0.648 (0.724)	**0.844**							
LWB	0.540 (0.334)	0.530 (0.436)	0.668 (0.665)	0.584 (0.601)	0.472 (0.435)	0.554 (0.610)	0.564 (0.568)	0.497 (0.348)	**0.890**						
PJ	0.583 (0.391)	0.663 (0.712)	0.624 (0.716)	0.796 (0.843)	0.593 (0.596)	0.689 (0.790)	0.630 (0.673)	0.685 (0.707)	0.631 (0.679)	**0.850**					
PWB	0.539 (0.423)	0.615 (0.545)	0.518 (0.481)	0.669 (0.800)	0.514 (0.486)	0.480 (0.538)	0.584 (0.545)	0.698 (0.699)	0.587 (0.548)	0.673 (0.731)	**0.847**				
SB	0.764 (0.616)	0.604 (0.574)	0.713 (0.549)	0.498 (0.532)	0.646 (0.736)	0.580 (0.573)	0.708 (0.666)	0.502 (0.594)	0.604 (0.483)	0.581 (0.457)	0.476 (0.567)	**0.843**			
SC	−0.610 (0.511)	−0.710 (0.557)	−0.583 (0.538)	−0.728 (0.682)	−0.655 (0.648)	−0.626 (0.636)	−0.655 (0.586)	−0.743 (0.711)	−0.549 (0.471)	−0.744 (0.670)	−0.662 (0.622)	−0.586 (0.713)	**0.861**		
ST	0.599 (0.422)	0.640 (0.402)	0.540 (0.571)	0.627 (0.532)	0.616 (0.646)	0.612 (0.654)	0.658 (0.612)	0.730 (0.612)	0.545 (0.469)	0.714 (0.614)	0.608 (0.455)	0.545 (0.584)	−0.821 (0.786)	**0.866**	
WWB	0.627 (0.383)	0.625 (0.446)	0.689 (0.752)	0.627 (0.696)	0.553 (0.672)	0.636 (0.729)	0.627 (0.681)	0.611 (0.601)	0.758 (0.764)	0.701 (0.767)	0.705 (0.810)	0.649 (0.595)	−0.657 (0.694)	0.616 (0.634)	**0.854**

^5^Note: The number set in boldface is the square root of AVE; the correlation coefficient is in the parentheses; the HTMT value is in parentheses.

### 4.2. Results of the PLS analysis

**Fig 2:**
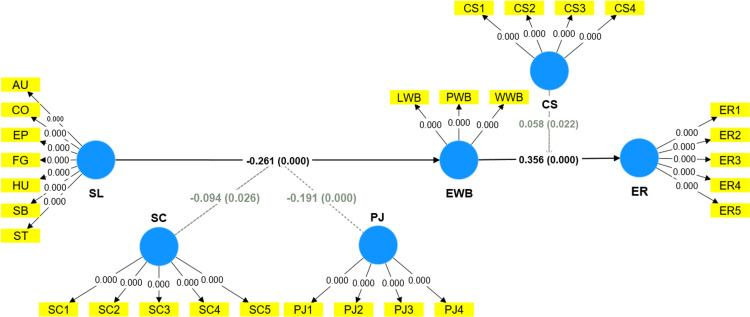
Results of the PLS-SEM analysis.

A two-stage approach for high-order construct analysis was adopted in this study. Dimensions were transferred into indicators for high-order construction (Fig 2). Table 5 shows the path coefficients and hypothesis results. The PLS analysis results of the model show that low servant leadership (*β* = −0.261, *t* = 5.397, *p *< 0.05) significantly leads to low employee subjective wellbeing. Hence, H1 is supported. Employee wellbeing (*β* = 0.356, *t* = 6.821, *p *< 0.05) significan*t*ly affects employee retention. Thus, H2 is supported. That is, lower subjective wellbeing caused by low servant leadership would reduce employee retention (*β* = −0.093, *t* = 3.885, *p *< 0.05). Thus, subjective wellbeing bridges low servan*t* leadership and employee retention.

**Table 5:** Hypothesis tests.

	Paths	Original sample	Sample mean	Standard deviation	t-statistic	P-values	Coincidence intervals		Test result
							2.50%	97.50%	
H1	SL → EWB	−0.261	−0.263	0.048	5.397	0.000	−0.353	−0.167	Supported
H2	EWB → ER	0.356	0.356	0.052	6.821	0.000	0.255	0.454	Supported
H3	SC x SL → EWB	−0.094	−0.169	−0.005	2.220	0.026	−0.173	−0.007	Supported
H4	PJ x SL → EWB	−0.191	−0.257	−0.118	5.110	0.000	−0.265	−0.117	Supported
H5	CS x EWB → ER	0.058	0.008	0.110	2.296	0.022	0.009	0.108	Supported
	SL - > EWB → ER	−0.093	−0.094	0.024	3.885	0.000	−0.143	−0.052	
	SC x SL → EWB → ER	−0.033	−0.033	0.016	2.026	0.043	−0.068	−0.002	
	PJ x SL → EWB → ER	−0.068	−0.067	0.016	4.284	0.000	−0.101	−0.039	

This study used R^2^ to reflect the explanatory power of the research model, indicating how the independent variable can explain the dependent variable [90]. R^2^ of dependent variables, such as employee wellbeing and retention, are 0.600 and 0.477, respectively, higher than the critical value requirements in R^2^ (weak: 0.25; medium: 0.50; strong: 0.75) [91]. Therefore, independent variables explain the dependent variables’ variances well, and the model setup is satisfactory overall.

**Fig 3:**
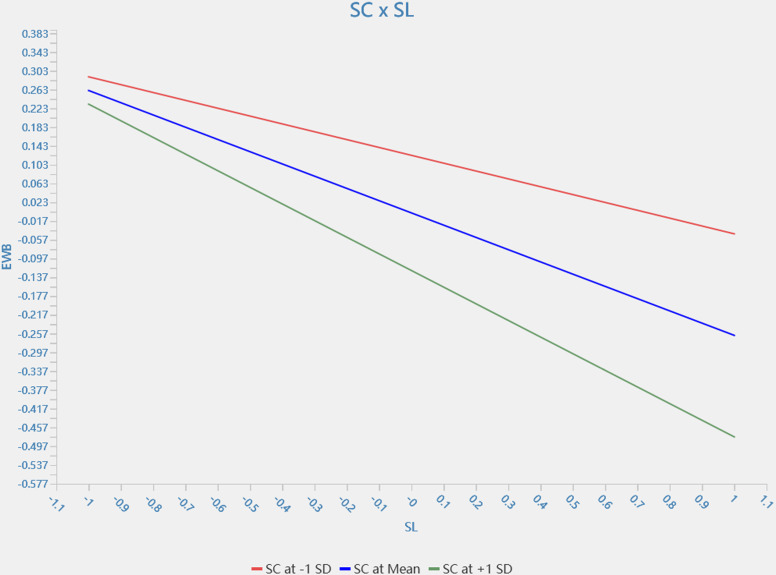
SC × SL.

**Fig 4:**
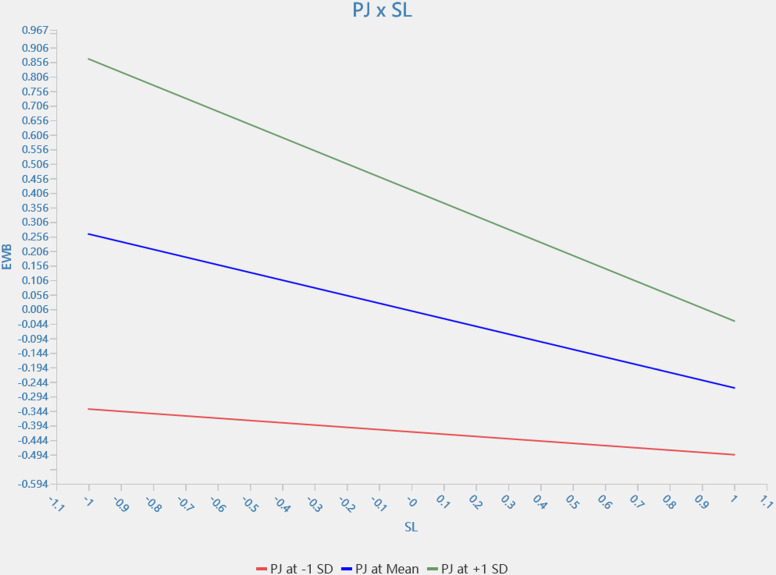
PJ × SL.

**Fig 5:**
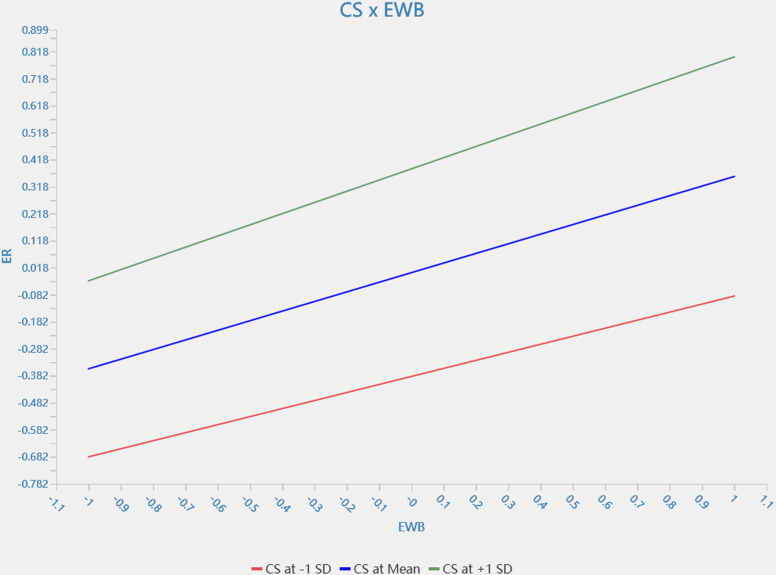
CS × EWB.

The results of the moderating effect show (Table 5 and Fig 2) that service climate moderates the relationship between servant leadership and employee wellbeing (β = −0.094, 95% *CI* = [−0.169, −0.005]). Additionally, procedural justice plays the same role as service climate in the relationship between servant leadership and employee well-being (*β* = −0.191, 95% *CI* = [−0.257, −0.118]). Customers’ satisfaction moderated the relationship between employee wellbeing and retention (*β* = 0.058, 95% *CI* = [0.008, 0.110]). These results indicate that H3, H4 and H5 are fully supported statistically. Figs 3–5 show simple slope analyses of three moderation effects.

## 5. Discussion

In the actual process of business operation, the top management decision often interferes with the frontline managers’ actual management behaviour [92–95]. Given the attention to the ubiquity of compliance with top management decisions, considerable scholarly work has linked to low servant leadership styles that affect employees’ job satisfaction, employee well-being and loyalty to service organisation [9,10]. Therefore, this study was conducted to reveal other disregarded but significant factors that may be constructive for employee retention. We demonstrate that servant leadership has an impact on employee well-being and retention (H1 and H2). We also predict the service climate and procedural justice influence of the low servant leadership on employees (H3 and H4). Low servant leadership is the consequence of inhibiting results from the top management decisions. Furthermore, customer satisfaction exhibits a relationship between employees’ wellbeing and retention (H5).

The main result is consistent with a previous study’s results [96]. It indicates a continuous logical relationship between servant leadership and employee well-being and retention. The dark side of leadership is mostly affected by top management’s low empathy, thereby preventing frontline managers from engaging in desirable ethical decisions [9]. Moreover, the top management’s decision (service climate and procedural justice) plays a positive role in mitigating the harmful effect of low servant leadership. Therefore, open and transparent communication between top management and frontline managers can help ensure that the latter understand the organisations’ goals and expectations. This case can reduce the likelihood of frontline managers feeling constrained by top management’s directives.

Service climate and procedural justice are internal elements of organisational operations and organisational intrinsic capabilities that they can control. Judging from the results of this research, a positive service climate and fair procedure execution can significantly moderate the impact of servant leadership on employee wellbeing. Therefore, this result appeals to astute managers to focus substantially on the capability of the organisational climate and company procedures, which can provide fairness. The two measures can awaken employee enthusiasm, thereby enhancing organisational performance. Accordingly, creating a supportive and inclusive organisational climate can help frontline managers feel significantly empowered to practice servant leadership. This goal can be achieved through such initiatives as team-building activities, recognition programmes and employee feedback mechanisms. Furthermore, ensuring that organisational procedures are fair and transparent can help enhance employees’ perception of procedural justice. Consequently, this situation can improve employee wellbeing and retention.

Additionally, customer satisfaction would also affect employee retention. It is out of the control of the service organisation, but it cannot be disregarded. Customer satisfaction has a significant impact on employee satisfaction and subjective wellbeing. Furthermore, when employees lack job wellbeing and support from their frontline managers, it can affect their retention, ultimately leading to quitting [71].

Employee wellbeing positively impacts employee retention, thereby partially supporting the results of Gordon et al. [97]. However, our results reveal that low servant leadership diminishes this positive outcome. Service employees are markedly valuable and attached to their health and well-being if the leader supports them. Moreover, positive service climates and procedural justice can compensate for low servant leadership, thereby supporting employee retention. Frontline managers should substantially focus on these issues and avoid making the wrong management decisions, particularly blind compliance with top management strategic decisions.

Besides the negative outcomes of servant leadership, we contribute role theory, highlighting that frontline managers who follow top management advice and instructions are likely to reduce servant leadership behaviour [9,10]. Little is known about frontline manager traits and predicting their management outcomes [32]. Many scholars have overlooked ‘the dilemma of servant leadership’. To fill in this gap, our study examines servant leadership, especially the pressure that top management exerts on frontline service leadership. Pressure from top managers on frontline servant leaders to change their leadership behaviours and management styles significantly affects junior employees at work. Additionally, antecedents inhibiting frontline managers from adopting servant leadership, such as distorted management ethics, insufficient service climates and lack of procedural justice, lead to low servant leadership, thereby negatively affecting employees’ well-being and retention. To avoid this situation, organisations should invest in leadership training programmes focusing on developing servant leadership skills. These programmes can help frontline managers understand the importance of servant leadership and provide them with the tools to practice it effectively. Additionally, organisations should regularly monitor and evaluate their leadership practices to identify areas for improvement. This strategy can involve conducting employee surveys, performance reviews and leadership assessments.

### 5.1. Theoretical implications

The theoretical implications emphasise the significant influence of service climate and procedural justice on servant leadership style. Although existing studies often emphasise the advantages of leadership [9], they tend to overlook the detrimental effects of insufficient servant leadership on service employees’ wellbeing and employee retention. Scholars must acknowledge the negative impact associated with low servant leadership and the consequent organisational challenges it poses. Studies have linked low servant leadership to employee wellbeing and employee retention [11], but studies addressing this phenomenon have been limited [7,31,32,41].

The result demonstrates that service climate and procedural justice facilitate servant leadership. This study offers insights into top management and the relationship between frontline managers and servant leadership. Although no direct evidence demonstrates that top management affects service climate and procedural justice, their attention to frontline managers on compliance with organisations’ decisions and operational process may distort their management style and leadership. Our findings contribute LMX and role theories and suggest a distinct feature for explaining the low servant leadership impact by adopting role theory for discussion. Role theory discusses the top management influence and goes beyond the common conceptualisations inhibiting frontline managers [9,41]. Theory integration provides a benchmark for further investigating how management operation affects employees’ well-being and retention rather than solely focusing on organisational performance [98].

### 5.2. Practical implications

By revealing the relationship amongst servant leadership, employee well-being and retention, as well as the moderating role of service climate, procedural justice and customer satisfaction, this study provides hotel managers with a guideline reference for management practices.

Firstly, the result indicates that service climate and procedural justice can impact servant leadership on employee wellbeing and retention. In some ways, a poor service climate and unfairness undermine employees’ sense of wellbeing and contribute to employee turnover. Top management compliance with organisation decisions and direction influences frontline managers’ work behavior, thereby leading to an unconstructive leadership style. Therefore, this research highlights the importance of servant leadership and underscores the need for top management to communicate more effectively with frontline managers, aligning their practices with employees’ expectations. Hotel managers can create a positive service-oriented service climate through team building activities, employee recognition programmes or ongoing communication. Additionally, frontline leaders should be consulted regularly and involved in important decisions. A transparent and fair decision-making process must be established to ensure that frontline leaders have a sense of fairness and involvement in their work. Top managers should lead by example, particularly by demonstrating servant–leadership behaviours and setting an example for frontline leaders. We suggest that top management provides flexibility to frontline managers. Moreover, top management ensures they will not sacrifice frontline managers to pursue organisational goals. Our finding is that practitioners need to be aware that when frontline managers are low in perceptive–taking perceived top management decisions–they are likely to disregard service climate and procedural justice, excluding servant leadership from their behavioural repertoire. They may be susceptible to the adverse effects of top management perception. We suggest that organisations set criteria for selecting the right person for leadership or management positions.

Secondly, the results confirm that enhancing employee psychological wellbeing bridges servant leadership and employee retention, making it a necessary and effective way to improve retention in organisations. Hotel managers can offer flexible working arrangements to their employees to maintain their work–life balance. Moreover, employees must be provided with opportunities for career development paths, psychological counselling and stress management training, thereby enhancing their subjective wellbeing. If enterprises can take effective measures for the psychological wellbeing of employees and create organisational attachment, then identification will be created. This situation can improve employees and maintain the best talents for organisations.

Lastly, customer satisfaction significantly impacts employee satisfaction and wellbeing in organisations [71]. Practitioners need to monitor and improve customer satisfaction, which remains an important task for the hospitality industry to continue promoting daily. Hotel managers can establish effective customer feedback channels to collect and process customer opinions in a timely manner and continuously optimise services. By focusing on customer satisfaction with our products and services, we can immediately identify any issues. Additionally, giving employees additional authority to solve customer problems on the spot can also enhance the customer experience. This situation enables us to take immediate action to improve employee wellbeing and reduce their likelihood of leaving.

### 5.3. Limitations and further studies

This research has several limitations. Firstly, data were collected only from the Chinese hospitality industry and may not be generalisable in other cultural contexts. The Chinese culture emphasises collectivism and relationship orientation, which may have a unique impact on the practice and effectiveness of servant leadership [99,100]. For example, Chinese employees may be markedly inclined to accept authoritative leadership, which may conflict with the democratic and empowering style of servant leadership. Therefore, future research could consider conducting similar studies in different cultural contexts to verify the generalisability of the findings. Secondly, respondents from the hospitality sector have limitations in this study. The research sample mainly focused on frontline employees in the hospitality industry. In future research, scholars should consider including employees at different levels from different industries to provide considerable credibility to the study. Thirdly, we suggest that future research can further examine the top management managerial decision affecting frontline managers’ psychological wellbeing associated with their trust in organisations. The leadership style can extend to other negative leadership and provide insight to the further study.

## Supporting information

S1 Appendix The original data of this study. https://doi.org/10.6084/m9.figshare.28606742.v1.
(XLSX)
